# Human error identification and risk assessment in loading and unloading of petroleum products by road trucks using the SHERPA and fuzzy inference system method

**DOI:** 10.1016/j.heliyon.2024.e34072

**Published:** 2024-07-04

**Authors:** Mostafa Mirzaei Aliabadi, Iraj Mohammadfam, Samane Khorshidikia

**Affiliations:** a Center of Excellence for Occupational Health, Occupational Health, and Safety Research Center, School of Public Health, Hamadan University of Medical Sciences, Hamadan, Iran; b Department of Ergonomics, Health in Emergency and Disaster Research Center, University of Social Welfare and Rehabilitation Science, Tehran, Iran; c Occupational Health and Safety Research Center, Occupational Health Engineering, School of Public Health, Hamadan University of Medical Sciences, Hamadan, Iran

**Keywords:** Human error, Loading operation, Unloading operation, Road truck, SHERPA, Fuzzy inference system (FIS)

## Abstract

Human error constitutes one of the primary causes of accidents, particularly in the context of loading and unloading operations involving road trucks, especially those carrying petroleum products. The process of identifying and evaluating human errors within these operations involves several key steps. Initially, all sub-tasks associated with loading and unloading are meticulously identified and analyzed utilizing Hierarchical Task Analysis (HTA), achieved through direct observation, document examination, and interviews. Subsequently, potential human error modes within each task are delineated using the Systematic Human Error Reduction and Prediction Approach (SHERPA). Finally, essential data for determining the criticality, probability, and severity of each error are gathered through expert elicitation and the application of Fuzzy Inference Systems (FIS).

Through the analysis of SHERPA worksheets, a total of 37 errors during loading operations and 14 errors during unloading operations of petroleum products were identified. Among these errors, the predominant category during loading operations was action errors, comprising 31 instances, while communication errors were the least frequent, occurring only twice. Similarly, action errors were most prevalent during unloading operations, constituting 13 instances. These errors were further categorized and ranked based on their risk levels, resulting in 27 levels for loading operations and 12 levels for unloading operations.

The consistent occurrence of action errors underscores the need for implementing control measures to mitigate their frequency and severity. Such strategies may include periodic training sessions to reinforce proper work procedures and the development of monitoring checklists, among other interventions.

## 1 Introduction

In the petroleum industry, ensuring safety is crucial due to the potential severe impacts on human life and the environment resulting from accidents. Establishing comprehensive safety measures is essential to ensure the safe and efficient operation of a refinery. Consequently, a critical objective is to minimize or eliminate risks in various areas of the refinery, including loading terminals [1]. Loading terminals play an important role in receiving petroleum products from both domestic and foreign oil refineries, and subsequently distributing them by tank trucks to meet industrial and individual needs. The distribution process involves loading the products into tanks of truck trucks, transporting them, and unloading them at the destination tanks [2,3].

The transportation of petrochemicals by road truck is a common practice globally. However, it is important to note that this activity carries inherent risks and can be a significant factor contributing to major accidents [4,5]. Not paying attention to the loading threshold, overfilling the tank, truck spill, rupture of the filling and unloading pipe, not paying attention to the equipment used when pumping fuel to the truck/unloading tank, followed by fire and explosion during loading and unloading of the truck have occurred, which has increased awareness of safety issues in the transportation of products by road trucks [1,6]. Statistics show that at least 2 major fires occur in oil refineries and loading terminals in the worldwide every year, and these incidents mostly occur in petroleum product loading platforms [7]. According to the International Truck Owner Pollution Federation (ITOPF) report, it was determined that there is the highest probability of explosion and fire during loading and unloading operations [8]. The analysis of incidents over two years of release of petroleum products in seven American states by the Agency for Toxic Substances and Disease Registry (ATSDR) showed that out of a total of 1369 incidents related to petroleum products, 512 injuries and 36 deaths were recorded [9]. Advances in petroleum industry programs may have significant social and economic benefits. However, Risks in the transportation of petroleum products have potentially devastating effects on the natural environment, vulnerable ecosystems, and problems associated with recovery and clean-up operations [[10], [11], [12], [13]]. Before 1956, there were limited laws to protect the control of contamination from petroleum products. These included the Refusal Act of 1899, the oil Pollution Act of 1924, and the Truck Act. However, over time, the importance of protecting natural and human resources to prevent pollution of petroleum products became more apparent [14].

Since some of the main steps of loading and unloading operations are still carried out by operators, special attention should be given to potential human errors during such operations. Human errors play a significant role in the majority of accidents that occur in the petrochemical industry [[15], [16], [17]]. Statistics recorded by the National Toxic Substances Incident Program (NTSIP) in the United States, showed that more than 40 % of petroleum accidents are caused by human practices, and human error is the most common cause of these accidents [9,18]. Ignoring the human element in the workplace not only result in loss of human performance, but also increases in the number of injuries and harm, which leads to significant financial losses [19,20]. Accordingly, anticipating and preventing potential human errors in critical operations conducted in the petrochemical industry is of paramount importance.

The Systematic Human Error Reduction and Prediction Approach (SHERPA), is one of the most practical methods for error classification in identifying valid errors related to a sequence of human activities. SHERPA belongs to a family of human error identification tools that have a psychological approach [[21], [22], [23]]. In their studies, Kirwan (1998) and Stanton (2006) mentioned the relative advantage of the SHERPA technique as ranking highest overall in a comparative study among human error detection techniques, including HEART, THERP, SLIM, and CREAM techniques, based on different performance criteria. Some advantages of this method include its ease of implementation and short execution time, determining the level of risk, identifying the consequences of errors, and ultimately providing control measures [24,25]. This systematic approach for predicting human errors was introduced by Embrey in 1986 [26] and is one of the most effective methods in various safety conditions, including the chemical industry [27], petrochemical [[28], [29], [30]], surgery [31,32], aviation [33,34], etc.

However, despite the high capability of the SHERPA technique in identifying and predicting human errors, it has not been able to quantitatively assess the risks associated with these errors and requires integration with other methods. The utilization of uncertain knowledge and subjective judgment creates a significant challenge in the application of this qualitative technique [35]. Uncertain knowledge arises due to a lack of awareness or insufficient awareness of the work process, the modifications in industrial methods and processes, and unreliable information. Using decision-based fuzzy models provides a solution for such a challenge. Fuzzy models based on the decision-making system can be mentioned as fuzzy AHP techniques, fuzzy TOPSIS, and FIS MATLAB tools. Studies have shown that the FIS method is superior to other methods based on decision-making systems in terms of validation and reduction of computing time. FIS is robust when dealing with uncertain, imprecise, and qualitative information, especially in situations where there is ambiguity surrounding a subject [[36], [37], [38]]. The probabilities and severities employed in the risk assessment process possess a certain level of uncertainty. By incorporating a fuzzy logic system, this methodology ensures logical consistency in the application of the conventional qualitative risk matrix approach by the risk assessor, achieved through the implementation of a fuzzy rule base. Based on the mentioned content, the objective of the current research is to identify and assess human errors in the loading and unloading operations of petroleum products by road trucks using the SHERPA and FST techniques.

## 2 Methods

**Fig. 1:**
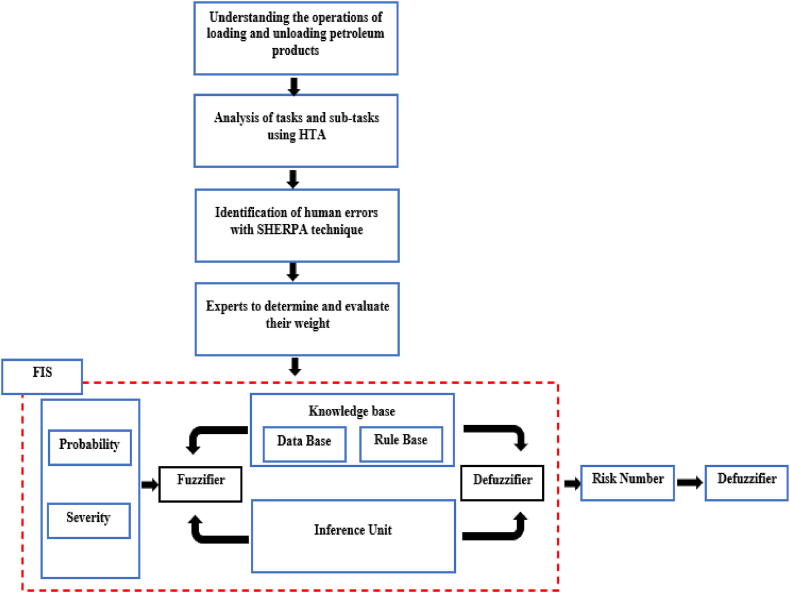
The proposed methodology.

This descriptive cross-sectional study was conducted at the loading terminal and fuel station of a petroleum products distribution company in Hamadan, Iran. The methodology employed in the study consists of three main phases (Fig. 1).

Phase 1: Identification of human errors in loading and unloading operations using the SHERPA technique.

Phase 2: Estimation of the risks associated with the identified errors using FIS.

Phase 3: Prioritization of the identified errors based on their risk numbers.

### 2.1 Human error identification

The process of human error identification involves identifying all the tasks and sub-tasks associated within the loading and unloading operations of petroleum products. This phase includes regular and frequent observations of the operations, reviewing working conditions, and analyzing past data to gain a comprehensive understanding of the operation. The hierarchical task analysis (HTA) technique is then used to divide the tasks and sub-tasks into work components. Upon drawing the HTA, the SHERPA technique is employed to identify the types of human errors in each subtask. Table 1 demonstrates the categorization of human errors into five categories: action errors, checking errors, retrieval errors, communication errors, and selection errors [39,40]. Using the SHERPA technique, possible errors are listed for each stage of the HTA. After determining the error modes, outcome analysis, error recovery analysis, error probability analysis, and error severity analysis are performed based on the SHERPA worksheet [41]. Additionally, a FIS is utilized to determine the probability and consequences of each error, and suggestions for reducing each error are provided. Finally, the error modes are quantified in terms of associated risk.

**Table 1:** Categories of human errors in SHERPA technique [39].

Error category	Code	Error mode
Action errors	A1	Operation too long/short
	A2	Operation mistimed
	A3	Operation in wrong direction
	A4	Too little/much operation
	A5	Misalignment
	A6	Right operation on wrong object
	A7	Wrong operation on right object
	A8	Operation omitted
	A9	Operation incomplete
	A10	Wrong operation on wrong object
Checking errors	C1	Check omitted
	C2	Check incomplete
	C3	Right check on wrong object
	C4	Wrong check on right object
	C5	Check mistimed
	C6	Wrong check on wrong object
Retrieval errors	R1	Information not obtained
	R2	Wrong information obtained
	R3	Information retrieval incomplete
Communication errors	I1	Information not communicated
	I2	Wrong information communicated
	I3	Information communication incomplete
Selection errors	S1	Selection omitted
	S2	Wrong selection made

### 2.2 Expert elicitation

To calculate the risk level of the identified errors in the loading and unloading of petroleum products, information regarding the probability of occurrence and the severity of the consequences of these errors is required. However, due to the limited available knowledge and the absence of documentation and records on the probability and occurrence of human errors, as well as the uncertainty inherent in human verbal judgments, additional methods are utilized.

Fuzzy logic and expert elicitation techniques are employed to address these challenges [42,43]. A carefully selected group of experts with diverse experiences, relevant expertise in the field of human error and operations of loading and unloading petroleum products, varying ages, and educational backgrounds play a crucial role in providing their judgments. These experts evaluate the probability of occurrence and severity of the identified errors based on their extensive knowledge in the field. Based on Table 2, each expert is assigned a specific score for each characteristic, considering five specific characteristics obtained from the experts. The job position is assigned a score ranging from 1 to 5, the years of work experience receive a score ranging from 1 to 5, the educational qualifications are given a score ranging from 1 to 5, and the age of the expert is considered for a score ranging from 1 to 4. Each expert accumulates scores for these five characteristics, resulting in a sum of five values for each expert. The weight coefficient for each expert is determined using the following formula:.

$${WC}_{Expert}=\frac{\sum_{j=1}^{5}S_{Expert,j}}{(\sum_{Expert=1}^{5}\left(\sum_{j=1}^{5}S_{Expert}\right)}$$

**Table 2:** Weighting criteria of different experts.

Constitution	Classification	Score
Professional position	Professor, Chief engineer, Director	5
	Assistant professor, Manager, Factory inspector	4
	Engineer, Supervisor	3
	Technician, Foreman	2
	Operator	1
Professional experience (year)	Up to 30	5
	20 to 30	4
	10 to 19	3
	6 to 9	2
	<6	1
Educational degree	PhD	5
	MSc	4
	BSc	3
	Higher national diploma	2
	High school	1
Age (year)	Up to 50	4
	40 to 50	3
	30 to 39	2
	<30	1

Explanation:

The weight coefficient(${WC}_{Expert}$)for each expert is calculated by dividing the sum of scores ($S_{Expert,j}$) for each feature by the total sum of scores for all features across all experts. This normalization ensures that the weight coefficients reflect the relative importance of each expert's scores within the overall context. The numerator captures the individual expert's scores for all features, while the denominator represents the total sum of scores for all experts and all features. This formula provides an effective method for deriving the weight coefficients, enabling robust analysis and decision-making processes based on the aggregated scores of the features. By utilizing this approach, the expertise and qualities of each expert are effectively integrated into the risk assessment process, leading to a more accurate evaluation of the risk level associated with the identified errors in the loading and unloading of petroleum products.

The risk assessment matrix employs five fuzzy language variables with linguistic terms, including “very low (VL)," “low (L)," “medium (M)," “high (H)," and “very high (VH)," to classify the probability and severity of the consequences associated with identified errors (Table 3). The severity of consequences and frequency are categorized and scaled based on the specific activity or processes being evaluated, taking into account the nature of the risks involved.

**Table 3:** Definition of fuzzy and crisp ratings.

Factors	Linguistic term(fuzzy set)	Crisp rating	fuzzy ratings
Probability	High (VH) Very	5	(4,5,5)
	High (H)	4	(3,4,5)
	Medium (M)	3	(2,3,4)
	Low (L)	2	(1,2,3)
	Very low (VL)	1	(1,1,2)
Severity	High (VH) Very	5	(4,5,5)
	High (H)	4	(3,4,5)
	Medium (M)	3	(2,3,4)
	Low (L)	2	(1,2,3)
	Very low (VL)	1	(1,1,2)
Risk	Very High (VH)	4	(15,20,25,25)
	High (H)	3	(10,15,20)
	Medium (M)	2	(5,10,15)
	Low (L)	1	(0,0,5,10)

For simpler risk assessments, a 3 × 3 cells matrix can be utilized, while larger structures like process plants may require a 5 × 5 or even a 7 × 4 matrix. In this particular study, a 5 × 5 cells risk matrix is recommended, indicating the presence of five distinct levels for both probability and severity of consequences.

The relationship between frequency, severity, and risk categories is determined by risk-based engineering rules. These rules establish the correlation between different levels of frequency and severity, enabling the assignment of appropriate risk categories.

In conclusion, the fuzzy ratings in the risk assessment matrix are determined by categorizing and scaling the severity of consequences and frequency, utilizing a 5 × 5 cells risk matrix, and applying risk-based engineering rules to establish the relationship between frequency, severity, and the corresponding risk categories [44,45].

### 2.3 Fuzzy inference system

FIS are based on a combination of fuzzy if-then rules, which assign fuzzy inputs to fuzzy outputs [46]. FIS is a method rooted in traditional logical reasoning using 0 and 1, and it was initially proposed by Zadeh in 1965. In 1975, Mamdani and Assilian applied fuzzy logic and fuzzy reasoning to control a steam engine, demonstrating its practical use in real-life applications. Since then, fuzzy theory has found wide application in industrial processes, including the petroleum industry [47]. There are various fuzzy combination methods available for fuzzy inference in the Mamdani fuzzy model. In this study, the max-min Mamdani combination method was employed [46,48]. Subsequently, the center of area (COA) calculation method was used for the defuzzification process, which converts fuzzy sets into crisp values [49,50].

In this study, FIS was utilized to analyze the criticality of the identified errors by determining the risk number associated with each error. This involved combining the probability of occurrence of the error with the severity of the damage caused by the error.

### 2.4 Application of the methodology: a case study

**Fig. 2:**
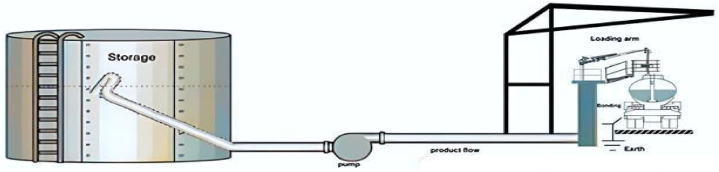
Petroleum product loading operations.

Road trucks are vehicles used worldwide for transporting petroleum products in the petrochemical industry. For the purposes of this study, the loading operations of petroleum products by road trucks at a loading terminal, which serves as a distributor of various petroleum products such as gasoline, gas oil, and kerosene, and manages fuel stations, have been considered. The loading terminal consists of 10 gasoline loading platforms, 10 gas oil loading platforms, and 4 kerosene loading platforms. Each loading platform associated with a specific product is identified by a distinctive color, with red indicating gasoline loading platforms, yellow indicating gas oil loading platforms, and blue indicating kerosene loading platforms. Fig. 2 illustrates a visual representation of a loading platform.

**Fig. 3:**
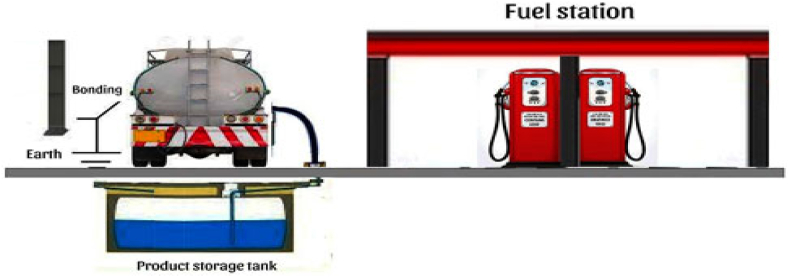
Petroleum product unloading operations.

The unloading operations are also considered at a fuel station located in Hamadan city. This fuel station is equipped with four 60,000-L tanks for petroleum products, consisting of two tanks for gasoline and two tanks for gas oil. The operational staff at this fuel station consists of six individuals. The unloading of petroleum products is carried out by road trucks, facilitated by the station's personnel (Fig. 3).

#### 2.4.1 Human error identification

Based on observations of work operations, worksite analysis, expert opinions, and literature review, a HTA was conducted for the loading and unloading operations of petroleum products. Table 4 provides details of the HTA for the loading operation, which consists of six sub-sections. Sub-sections 1 to 6 respectively describe the following tasks: checking the documents of the truck driver, preparing the truck for loading, loading of petroleum products, conducting quality control, sealing oil trucks, and checking driver information and issuing forms. The sub-section “preparing the truck for loading” is further divided into three tasks, while “sealing” is divided in to seven tasks. Additionally, the task of washing the truck is divided into 12 sub-tasks, and the task of sinking the truck is divided into seven sub-tasks.

**Table 4:** HTA petroleum product loading operation.

Task step	Task description	Plan
**1**	**Check the documents of the truck driver**	Check the cargo transport permit, driver's license, truck documents, and record the information
**2**	**Preparing the truck for loading**	
2.1	Placement of the truck in the loading line	- Place the truck in the designated route for loading, as indicated by markings and guide signs. - Place the truck parallel or perpendicular to the loading line and maintain a suitable distance from other trucks.
2.2	Check the truck bill of lading and record information	- Verify the truck's bill of lading to ensure that the truck aligns with the specified cargo or product mentioned in the bill. - Record the relevant information from the bill of lading in the logbook
**2.3**	**The oil tank is washed**	
2.3.1	Place the truck in the washing area	Drive the truck to the designated washing area and place it at the specified location within the washing zone.
2.3.2	Turn off and pull the handbrake	- Switch off the engine of the truck. - Engage the handbrake to secure the truck
2.3.3	Connecting the earth connection cable to the truck	Attach the earth connection cable to the appropriate grounding point of the truck.
2.3.4	Loading volume of 400-300 L of kerosene	Fill the tank with 400-300 L of kerosene, taking into account the capacity and requirements of the tank.
2.3.5	Move the truck for 5 min in the specified direction	Start the engine and drive the truck in the specified direction within the washing area for a duration of 5 min, allowing the kerosene to mix with the tank during the truck's movement, effectively cleaning the tank.
2.3.6	Placement of the truck in the unloading platform	After the 5-min movement for cleaning the truck's tanks, drive the truck to the designated unloading platform for the disposal of the kerosene in the tanks.
2.3.7	Turn off and pull the handbrake	- Switch off the engine of the truck. - Engage the handbrake to secure the truck
2.3.8	Connecting the earth connection cable to the truck	Attach the earth connection cable to the appropriate grounding point of the truck.
2.3.9	Connect the drain pipe to the tank	Connect the drain pipe sequentially to each tank on the truck containing kerosene for cleaning purposes, allowing the effective discharge of kerosene from the tanks.
2.3.10	Open the drain valve	Carefully open the drain valve of each tank that the drain pipe is connected to, allowing the contents of the tank to be freely released through the drain pipe.
2.3.11	Disconnect the drain pipe from the tank	Disconnect the drain pipe from the tank's outlet once the draining process is complete.
2.3.12	Close the drain valve	After completing the discharge and disconnecting the drain pipe, close the drain valve on the truck's tank.
**2.4**	**Removing the products remaining in the tanks of oil trucks**	
2.4.1	Placement of the truck in place	The driver should place the truck in the designated place to remove the remaining products from the truck.
2.4.2	Turn off and pull the handbrake	- Switch off the engine of the truck. - Engage the handbrake to secure the truck
2.4.3	Connecting the earth connection cable to the truck	Attach the earth connection cable to the appropriate grounding point of the truck.
2.4.4	Connect the drain pipe to the truck tank	Connect the drain pipe to each tank of the truck in sequence to drain the tanks effectively.
2.4.5	Open the drain valve	Carefully open the drain valve of each tank that the drain pipe is connected to, allowing the contents of the tank to be freely released through the drain pipe.
2.4.6	Separate the drain pipe from the tank	Disconnect the drain pipe from the tank's outlet once the draining process is complete.
2.4.7	Close the drain valve	After completing the discharge and disconnecting the drain pipe, close the drain valve on the truck's tank.
2.5	Operator awareness of the truck entering the loading platform	The truck's entry to the loading platform is communicated to the operator of the platform.
**3**	**Loading of petroleum products**	
3.1	Placement of the truck on the loading platform	The truck is placed in the specified loading platform.
3.2	Parking the truck by the driver on the loading platform	- The driver aligns the truck with the designated parking area on the loading platform. - The driver ensures the truck is properly positioned and stationary on the loading platform.
3.3	Turn off the truck and pull the hand brake and place the wedge next to the tires	- Switch off the engine of the truck. - Engage the handbrake to secure the truck. - The driver places wedges under the tires to prevent sudden movement of the truck.
3.4	Presence of firefighter next to the loading platform	The firefighter is placed next to the loading platform to prevent accidents.
3.5	Check the bill of lading	- Verify the truck's bill of lading to ensure that the truck aligns with the specified cargo or product and matches the type of fuel required by the loading platform. - Record the relevant information from the bill of lading in the logbook.
3.6	Check the truck drain valves by the loading operator	All truck drain valves are inspected by the loading operator to ensure that no residue is present in the tank.
3.7	Connecting the earth connection cable to the truck	Attach the earth connection cable to the appropriate grounding point of the truck.
3.8	Open the truck tank lid	Open the truck's inlet tank lid for loading the petroleum cargo.
3.9	Inserting the loading arm into the truck tank	The loading arm is placed at the first inlet tank of the truck for loading the petroleum cargo.
3.10	Locking the loading arm	The loading arm lever is locked to prevent sudden detachment of the arm from the truck tank.
3.11	Setting the Volumetric device	the Volumetric device is adjusted according to the volume written in the bill of lading.
3.12	Pump the product into the tank	The start button of the volumetric device is pressed to pump the petroleum product into the tank.
3.13	Place the opening of the loading arm in the aluminum bucket	After loading the product, the loading arm lever is detached from the tank and placed inside an aluminum bucket attached to the loading arm.
3.14	Measurement of product level in tank by Brass Rod	The operator manually measures the level of the loaded petroleum product in the tank using a brass rod.
3.15	Close the loaded tank lid	The operator closes the truck tank lid, which contains the loaded petroleum product.
3.16	Separate the earth connection cable from the truck	After loading all the truck's tanks, disconnect the earth connection cable from the truck.
3.17	Loading data recording	Record all the information of the loaded content in the truck's tanks.
**4**	**Quality Control**	The information of the bill of lading and the loading performed by the quality control personnel is systematically recorded.
**5**	**Sealing oil trucks**	
5.1	Placement of the truck at the place of sealing	The truck is placed in the sealing area by the driver and parked.
5.2	Turn off and pull the handbrake	- Switch off the engine of the truck. - Engage the handbrake to secure the truck
5.3	Check documents and record information	The bill of lading information is checked by the sealing operator.
5.4	Receive numbered seals by the driver	Numbered seals are provided to the driver by the sealing personnel for securing the truck.
5.5	Product level measurement in the inlet tank by Brass Rod	To ensure the product level in the tank, the truck tanks are measured and checked by a brass rod.
5.6	Installation of seals on loading and unloading tanks	The sealing nuts are placed by the sealing operator on all the valves of the loading and unloading tanks of the truck.
5.7	Exit the truck from the sealing area	After installing all the sealing nuts, the truck is taken out of the sealing place by the driver.
**6**	**Check driver information and issuance form**	The truck and driver information is reviewed by the personnel in the exit section, and permission to exit is granted to the driver for the truck's departure from the loading terminal.

Similarly, the unloading operation of petroleum products is divided into two sub-sections: checking the driver's license and completing the product issuance and unloading form. These details are presented in Table 5.

**Table 5:** HTA petroleum product unloading operation.

Subtask	Task description	Plan
**1**	**Check driver documents**	Check the cargo transport permit, driver's license, truck documents, and record the information
**2**	**Product unloading**	
2.1	Oil truck park on the unloading platform	The truck is placed in the specified unloading platform.
2.2	Turn off the truck and pull the hand brake and place the wedge next to the tires	- Switch off the engine of the truck. - Engage the handbrake to secure the truck. - The driver places wedges under the tires to prevent sudden movement of the truck
2.3	Connecting the earth connection cable to the truck	Attach the earth connection cable to the appropriate grounding point of the truck.
2.4	Connect the drain hose to the truck	Connect the drain hose to each tank of the truck in sequence to drain the tanks effectively.
2.5	Open the drain valve	Carefully open the drain valve of each tank that the drain hose is connected to, allowing the contents of the tank to be freely released through the drain hose.
2.6	Disconnect the drain hose from the tank	Disconnect the drain hose from the tank's outlet once the draining process is complete.
2.7	Insert the drain hose into the aluminum bucket	After completing the discharge and disconnecting the drain hose, place the drain hose inside an aluminum bucket.
2.8	Separate the earth connection cable from the truck	After unloading all the tanks of the truck, disconnect the earth cable from the truck.
2.9	Record unloading information	The truck and driver information is reviewed by the personnel in the exit section, and permission to exit is granted to the driver for the truck's departure from the fuel station.

It states that each stage of the HTA analysis was conducted by a team of experts to preliminarily identify errors based on tasks, code errors, and error types separately for the loading and unloading operations. The SHERPA technique was utilized for this purpose, and Table 6, Table 7, Table 8 provide the relevant information. According to these tables, a total of 37 human errors were identified for the six sub-sections in the loading operation, while a total of 14 human errors were identified for the two sub-sections in the unloading operation. These findings are summarized in Table 9.

**Table 6:** Errors caused by HTA in loading operations using SHERPA.

Error no	Task step	Error mode	Error	Consequences	Recovery	Error reduction
1	1	C2	The check is incomplete	Wrong entry of the truck to the loading line	2.2	Existence of daily office to record all information of incoming trucks.
2	2.1	A3	The truck is moving in the wrong direction.	Disruption of the loading process	No recovery	The signage at the terminal should be easily visible from all directions.
3	2.2	A9	The check is incomplete	The truck is not washed.	3.5	Develop written work instructions
				The type of product for loading is not suitable for the type of truck. Placing the truck on the wrong platform		
4	2.3	A8	The washing of the oil tank is omitted.	There is a volume of the previous product in the truck which causes impurities in the loaded product.	3.5	Staff training
5	2.3	A9	The truck is washed incompletely.	Oil tank overflow during loading due to the presence of additional product from previous loading	3.5	Staff training
6	2.4	A8	Removal of the remaining product in the trucks is omitted.	Incompatibility of the type of product loaded with the product that was last loaded by the truck.	3.5	Staff training
7	2.4	A9	Removal of the remaining product in the truck is incomplete.	There is a volume of the previous product in the truck which causes impurities in the loaded product	3.5	Staff training
8	2.5	I2	Wrong information is given to the operator.	The truck enters the wrong loading platform	3.5	Provide wireless communication for each of the loading platforms.
9	3.1	A3	The truck was mistakenly placed on another loading platform.	The loading of the product type is not in accordance with the bill of lading	3.5	The loading platform number must be written in the bill of lading by the loading line operator.
10	3.2	A9	The truck was not properly parked.	Risk of the operator falling during loading operations due to the large distance between the loading arm and the truck	No recovery	Installation of Appropriate Signage
11	3.3	A8	The driver omitted to turn off the truck.	Risk of explosion during loading operation	No recovery	Installation of Appropriate Signage
12	3.3	A8	The driver is omitted to pull the hand brake.	Sudden movement of the truck during the loading operation and spilling of the product on the loading platform	No recovery	Installation of Appropriate Signage
13	3.3	A8	Wedge placement next to truck tires has been omitted.	Sudden movement of the truck during the loading operation and spilling of the product on the loading platform	No recovery	Installation of Appropriate Signage

**Table 7:** Errors caused by HTA in loading operations using SHERPA.

Error no	Task step	Error mode	Error	Consequences	Recovery	Error reduction
14	3.4	I1	The firefighter is not present next to the loading platform.	The firefighter was not notified.	No recovery	Staff training
						Develop written work instructions
15	3.5	C2	The check is incomplete	Truck on the wrong platform.	No recovery	Staff training
						Develop written work instructions
16	3.6	C1	Checking the drain valves is omitted.	The drain valves were open	No recovery	Staff training
						Develop written work instructions
17	3.7	A2	The earth connection cable is not connected to the truck at the required time.	Explosion risk in loading operations	No recovery	Installation of audible and visual alarms to ensure the installation of the earth connection cable to the truck.
18	3.8	A	Exit the loading arm from the tank before the loading is complete	Spraying of the product on the operator and the truck and the risk of fire and explosion	3.9	Audible alarm sound Volumetric device
						It should be large enough to be clearly audible to the operator.
19	3.8	A9	The loading arm is not completely in the tank.	Sudden exit Loading arm from the truck	3.9	Staff training
20	3.9	A8	Locking the loading arm is omitted.	Sudden exit Loading arm from the truck	No recovery	Staff training
21	3.10	A4	Setting the Volumetric device is more than the bill of lading volume.	Product overflow from the tank	No recovery	Attention to workers' work-rest periods
22	3.10	A4	Setting the Volumetric device is less than the bill of lading volume.	The loaded volume is less than the amount recorded in the bill of lading.	3.12	Attention to workers' work-rest periods
23	3.10	A8	Volumetric device setting is omitted.	Loading is done based on the information stored in the previous load on the volumetric device.	3.12	Attention to workers' work-rest periods
24	3.11	A8	putting up the opening of the loading arm in the aluminum bucket is omitted.	Spraying of product residue from the loading arm outlet onto the loading operator and truck	No recovery	Permanent connection of bucket to drain hose
25	3.12	A	The measurement of the product level in the tank is done earlier by Brass Rod.	Risk of explosion due to static electricity	No recovery	Staff training

**Table 8:** Errors caused by HTA in loading operations using SHERPA.

Error no	Task step	Error mode	Error	Consequences	Recovery	Error reduction
26	3.13	A8	Closing the loading tank lid is omitted.	Spraying of petroleum products while moving the truck	No recovery	Staff training
27	3.13	A9	The truck tank lid is not Completely closed.	Spraying of petroleum products while moving the truck	No recovery	Staff training
28	3.14	A2	Separation of the earth connection cable from the truck is done sooner.	Risk of fire/explosion during loading operations	No recovery	Installation of audible and visual alarms to ensure the installation of the earth connection cable to the truck
29	3.14	A8	The separation of the earth connection cable from the truck is omitted.	Damage to the earth connection cable during the movement of the truck	No recovery	Installation of audible and visual alarms to ensure the installation of the earth connection cable to the truck
30	3.15	A8	Loading recording data is omitted.	Error in the total amount of product loaded during the work shift	No recovery	Preparation of checklist
						Staff training
31	3.15	A9	Incomplete information is recorded.	Error in the total amount of product loaded during the work shift	No recovery	Preparation of checklist
						Staff training
32	4	A9	Incomplete information is recorded.	The information was not verified and matched correctly.	No recovery	Preparation of checklist
						Staff training
33	5.1	A9	Checking and recording incomplete bill of lading information is done	Delivery of wrong seals to the truck driver	No recovery	Staff training
34	5.2	A	The measurement of the product level in the tank is done earlier by Brass Rod.	Risk of explosion due to static electricity	No recovery	Staff training
35	5.3	A8	The sealing of loading and unloading tanks is omitted.	petroleum product theft.	No recovery	alarm system
				explosions and human casualties.		
36	5.3	A9	The lid of the loading and unloading tanks is not completely sealed.	petroleum product theft.	No recovery	alarm system
				explosions and human casualties		
37	6	C2	Checking the incomplete issuance Sheet is done	The truck driver is mistakenly allowed to leave.	No recovery	Staff training

**Table 9:** Errors caused by HTA in unloading operations using SHERPA.

Error no	Task step	Error mode	Error	Consequences	Recovery	Error reduction
1	1	C2	Checking documents is incomplete.	The truck enters the company by mistake	No recovery	Staff training
2	2.1	A9	The truck was not properly parked.	The connection between the drain hoses and The truck tank is not connected properly	No recovery	Develop written work instructions.
						Installation Suitable sign
3	2.2	A8	The driver omitted to turn off the truck.	The danger of sparks and explosions during unloading operations	No recovery	written work instructions.
						Installation of Appropriate Signage
4	2.2	A8	The driver is omitted to pull the hand brake.	Sudden movement of the truck during the discharging operation	No recovery	Develop written work instructions.
						Installation Suitable sign
5	2.2	A8	Wedge placement next to truck tires has been omitted.	Sudden movement of the truck during the discharging operation	No recovery	Develop written work instructions.
						Installation of Appropriate Signage
6	2.3	A2	The earth connection cable is not connected to the truck at the required time.	Risk of explosion during unloading operation	No recovery	Installation of audible and visual alarms to ensure the installation of the earth connection cable to the truck
7	2.4	A9	The unloading hose is not fully connected to the unloading tank.	Disconnect the unloading hose to the unloading tank	No recovery	Develop written work instructions
8	2.5	A	The unloading valve is open in a short time and the product is not removed completely.	Remaining the product in the tank of the truck	No recovery	Staff training
						Develop written work instructions
9	2.6	A2	Disconnect the unloading hose before closing the unloading valve.	Spraying the product during unloading	No recovery	Staff training
						Develop written work instructions
10	2.7	A8	Inserting the drain hose in the aluminum bucket is omitted.	Leakage of product in drain hose	No recovery	Permanent connection of bucket to drain hose
11	2.8	A2	Disconnecting the earth connection cable from the truck is done at the wrong time.	Danger of sparks and explosions during unloading	No recovery	Install audible or visual alarms to notify when the earth connection cable is connected or disconnected from the truck
12	2.8	A8	Disconnecting the earth connection cable from the truck is omitted.	The earth connection is interrupted while the truck is moving.	No recovery	Install audible or visual alarms to notify when the earth connection cable is connected or disconnected from the truck
13	2.9	A8	Record information is omitted.	Error in the total volume of product unloaded during the shift	No recovery	Attention to workers' work-rest periods
14	2.9	A9	Incomplete information is recorded.	Error in the total volume of product unloaded during the shift	No recovery	Attention to workers' work-rest periods

*Weighting coefficients.

**Master of Science.

***Bachelor of Science.

#### 2.4.2 Fuzzy risk assessment

In order to assess the risk associated with the identified errors, the experts were assigned weights based on Table 10. Subsequently, the experts ranked each error in terms of its probability of occurrence and the severity of its consequences using predetermined linguistic variables. Expert opinions on all identified errors can be found in Table 11, Table 12.

**Table 10:** Expert weighting coefficients.

Expert number	Job	Education level	Age	Work experience (Years)	Weight factor	WC_Expert_*
1	Operator	Higher national diploma	30–39	6–9	7	0.142
2	Operator	Higher national diploma	30–39	10–19	8	0.163
3	Chief Engineer	**MSc	30–39	10–19	14	0.285
4	Engineer	*** BSc	30–39	<6	9	0.183
5	Manager	Higher national diploma	40–50	6–9	11	0.224

**Table 11:**
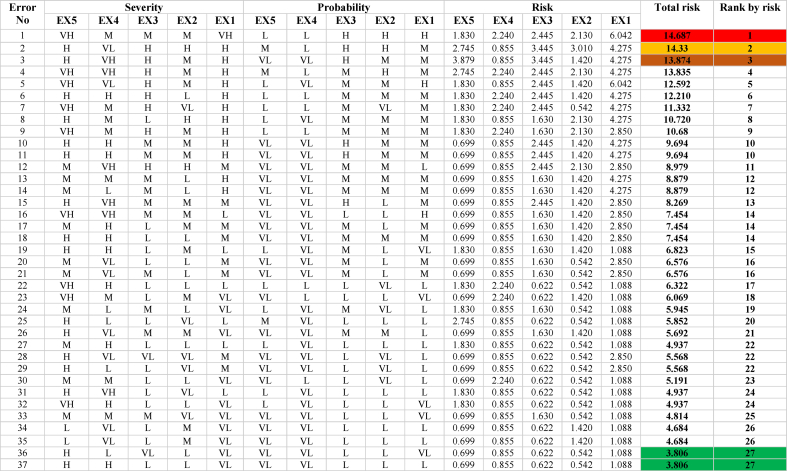
Probability and severity of all loading operation errors based on expert opinions.

**Table 12:**
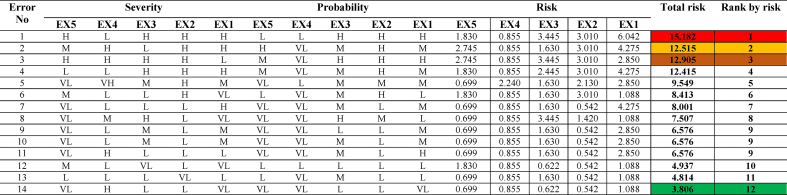
Probability and severity of all unloading operation errors based on expert opinions.

**Fig. 4:**
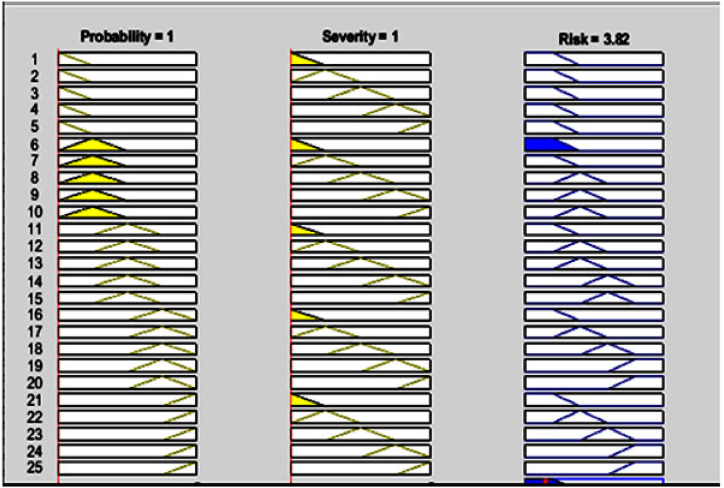
Graphic symbol of fuzzy reasoning structure.

**Fig. 5:**
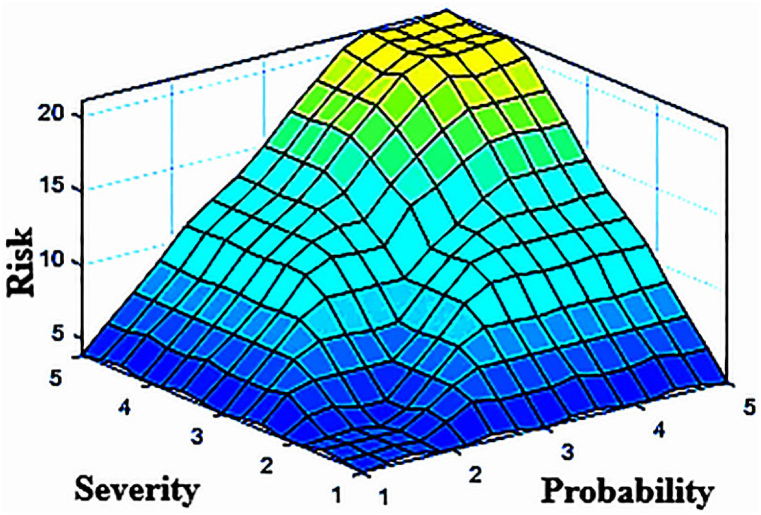
Control surface of FIS on severity and probability.

Then, if-then fuzzy rules were developed to represent the relationship between input and output variables based on the insights and expertise of the experts. The collected data, including the probability score and severity of the identified errors, were input into the FIS (Fuzzy Inference System) and analyzed by the fuzzy inference engine using the if-then rules. Through the defuzzification process, the fuzzy values obtained were converted into a risk number. The COA method was commonly used for defuzzification to establish the relationship between input and output variables. The structure of fuzzy reasoning for determining the risk number is illustrated in Fig. 4.This figure shows the fuzzy sets and its membership function for each variable used in the fuzzy risk assessment matrix.Fig. 5 depicts the interdependence of the probability and severity variables as a control surface in the FIS based on the defined rules. This figure shows the relationships between input variables (probability and severity) and the output variable (risk number) in the risk matrix. This three-dimensional graphic visually represents the level of risk. -. All calculations were performed using the commercial software MATLAB R2018b Finally, the errors were ranked based on the risk number. Table 11, Table 12 provide information on the total risks and the number of calculated risks for the loading and unloading of petroleum products. A low level of risk corresponds to a situation with a low probability of occurrence and low severity, whereas a high level of risk indicates a situation with a very high probability of occurrence and severity.

## 3 Results

**Fig. 6:**
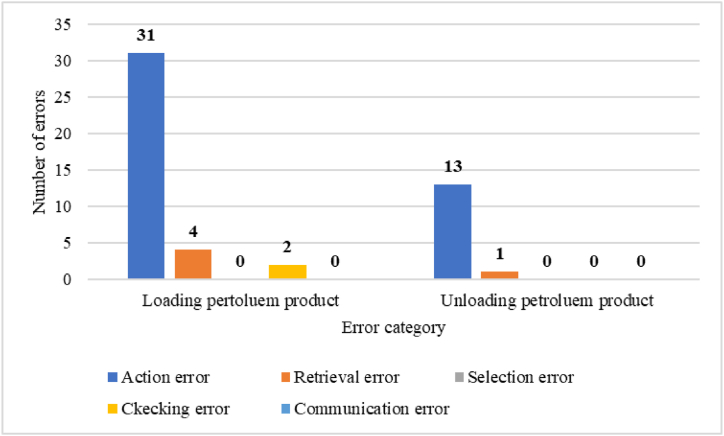
The number and categories of errors in loading and unloading operations.

In this study, a total of 37 human errors were identified in loading operations and 14 human errors in unloading operations through HTA analysis. Specifically, in loading operations, there were 31 errors categorized as action errors, 4 errors categorized as checking errors, and 2 errors categorized as communication errors. Similarly, in unloading operations, there were 13 action errors and 1 checking error. The number and categories of errors are visually presented in Fig. 6.

**Fig. 7:**
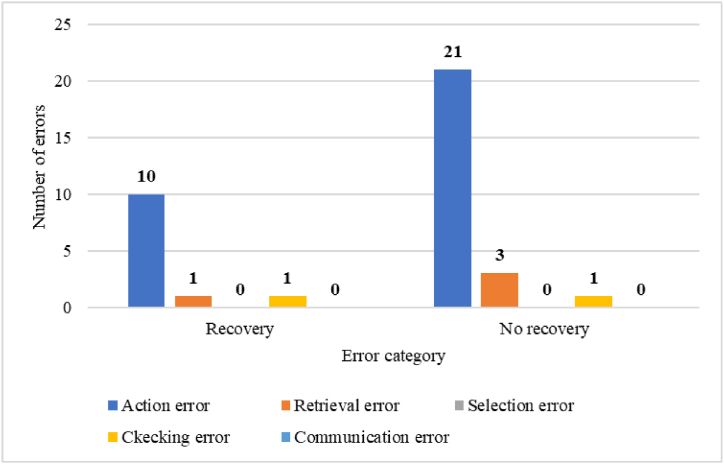
The number of errors in terms of recovery in petroleum product loading operations.

In the loading operation, most of the action errors occur in the form of performing the operation incompletely (A9) and omitted (A8). In checking errors, the check is usually performed incompletely (C2), and in communication error, information is not exchanged (I) or incorrect information is exchanged (I2). In the unloading operation, most of the action errors occur in the form of performing the operation omitted (A8). In checking errors, the check is usually performed incompletely (C2). Also, according to Fig. 7, in petroleum product loading operations, the number of unrecoverable errors included 21 action errors (56.75 %), 3 checking errors (8.10 %) and 1 communication error (2.70 %).

**Fig. 8:**
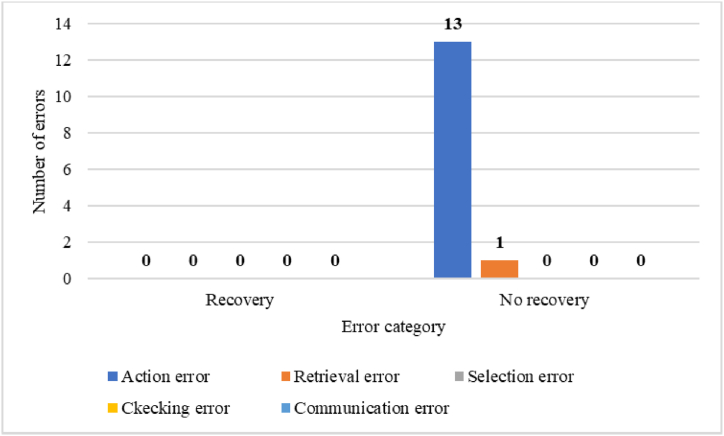
The number of errors in terms of recovery in petroleum product unloading operations.

In the unloading operation, 13 action errors (92.85 %) and 1 checking error (7.14 %) had no recovery (Fig. 8).

Observing the errors, it was found that in both operations, most of the errors are not effectively addressed or corrected, as per the definition of error recovery in the SHERPA technique, which entails taking actions in subsequent stages to restore the system to its initial state. Therefore, these errors were considered unrecoverable, as they could not be rectified or mitigated in the subsequent stages of the operations. Furthermore, a significant number of unrecoverable errors in both operations were identified as action errors.

Considering this definition, the identification of a significant number of unrecoverable errors in both operations highlights the failure to implement the necessary actions for error recovery. These errors, once they occurred, could not be effectively addressed or corrected in the subsequent stages, resulting in prolonged or irreversible consequences.

To address this issue and minimize the occurrence of unrecoverable errors, it is crucial to implement proactive measures, such as employee training programs, the utilization of checklists, installing audio and visual alarms, developing written work instructions, and paying attention to the work-rest period of employees. By implementing these measures, operators can improve the ability to recover from errors and prevent their escalation into unrecoverable situations.

Failure to implement these preventive measures can lead to severe consequences, such as explosions, petroleum product leakage, and failure to load or unload products according to the required volume. Therefore, it is imperative to prioritize the identification and mitigation of unrecoverable errors to enhance safety and operational efficiency in petroleum terminals. The risk assessment by the FIS showed that in the operation of loading petroleum products (Table 11), 27 ranks were determined for the risk level of identified errors. A number of errors also scored the same risk score. The highest risk score was related to error, checking the drain valves is omitted (error no 16). And the lowest risk score related to errors, removal of the remaining product in the truck is incomplete (error no 7) and exit the loading arm before the loading was complete (error no 18). Also, in the operation of unloading the petroleum product (Table 12), 12 ranks were set for the risk level of identified errors. The highest risk score related to the error, the disconnection of the earth connection cable from the truck is omitted (error no 12). And the lowest risk score for the error was the driver is omitted to turn off the truck (error no 3).

## 4 Discussion

Human error is a notable concern in various industries as it significantly impacts the occurrence of accidents. Additionally, a crucial strategy for improving safety performance in the petrochemical industry reducing human error [9,51]. Tt is crucial to understand that road truck transfers require significant operator engagement, occur frequently, and pose a considerable risk of serious accidents. As a result, the evaluation and management of human factors play a vital role in ensuring the safety and efficiency of these operations [4]. This study presents a human error evaluation method and applies it to petroleum product loading and unloading terminals. Initially, task analysis was performed hierarchically for both operations. Subsequently, the researchers completed the worksheets using the SHERPA technique.

In the petroleum product loading operation, the main errors identified were 31 action errors (83.78 %). checking errors were in the second place with the number of 4 errors (10.81 %) and communication errors were in the third place with the number of 1 error (5.4 %). There were no retrieval errors and selection errors in this operation. Similarly, in the oil product unloading operation, the main errors identified were 13 action errors (92.85 %). checking errors were also in the second place with the number of 1 error (7.14 %). Also, no retrieval errors, selection errors, and communication errors were detected. To reduce the identified errors, solutions such as employee training, preparing a checklist, installing audio and visual alarms, and paying attention to the work-rest period of employees were suggested. Similarly, the study conducted by Sabbaghpoor Azariyan et al., with the aim of identifying, analyzing and management of human errors in the filtration unit of the oil refinery showed that out of 181 identified errors, 154 errors were action errors, 24 errors were checking errors, and 2 errors were communication errors. And 1 error was one of the selection errors. No retrieval errors were detected. Also, to reduce the identified errors, employee training was suggested as a corrective solution [52,53].

SHERPA is considered as a comprehensive and robust technique for detecting and predicting human error. However, it is a qualitative method and has significant uncertainties. In this study, fuzzy set theory was used as a complementary quantitative approach for the assessment of the identified errors by the SHERPA technique. In this way, a three-dimensional risk envelope or surface is created and used to calculate risk values associated with errors.

According to Table 11 in the petroleum product loading operation, the three human errors that allocated the highest risk score are as follows: Checking the drain valves is omitted (14.68), The separation of the earth connection cable from the truck is omitted (14.33), and the sealing of loading and unloading tanks is omitted (13.87). In the petroleum product unloading operation, according to Table 12, 14 human errors were identified and ranked in 12 levels from high to low risk. The findings indicate that the three human errors that allocated the highest risk score are as follows: Disconnecting the earth connection cable from the truck is omitted (15.18), Disconnect the unloading hose before closing the unloading valve (12.90), and the earth connection cable is not connected to the truck at the required time (12.51).

One of the non-recovery errors in both loading and unloading operations was The earth connection cable is not connected to the truck at the required time, which could lead to an explosion and fire. Research showed that static electricity is very important when loading road trucks because of the load generated by the product flow through the pipeline.

The utilization of fuzzy sets is appropriate for managing the imprecision often associated with accident probability and severity data. The total number of rules required to build a fuzzy inference engine is the product of the number of rows and the number of columns for the qualitative risk matrix based on the probability and severity.

The integration of the SHERPA technique as a qualitative method and the fuzzy inference system as a quantitative method has proven highly effective in identifying and quantifying human errors with greater accuracy. While previous studies typically relied on a single probability factor to assess human error, our proposed approach incorporates two factors: the probability of human error and the severity of error occurrences. This integration allows for a more comprehensive and quantitative assessment, resulting in a well-organized ranking of errors and consideration of various priorities. By employing this approach to identify and prioritize the risks associated with human errors, organizations can allocate resources more efficiently for risk management, thereby improving safety and efficiency in petroleum product terminals.

Nonetheless, it's important to mention that this study didn't classify the identified error risks into acceptable and unacceptable levels, which could be an avenue for future research. Moreover, integrating specific criteria for risk categorization and addressing other aspects of human error management would be beneficial for further studies.

Additionally, one limitation of this study was the formation of an expert team consisting of only five specialists. We had to form an expert team with just five experts as only this number responded to our request to participate. One concern of this study was the potential for an unreasonable aggregation due to the limited number of experts. It's suggested that future studies employ the large-scale group method, in which a sufficient number of experts provide their fuzzy rankings in linguistic values. This method fosters effective coordination and collaboration among experts' mindsets, enhancing accuracy in assessing human error [54].

## 5 Conclusion

In conclusion, this study focused on the evaluation of human errors in petroleum product loading and unloading terminals using a combination of qualitative and quantitative methods. The SHERPA technique was employed for error identification and ranking, while fuzzy set theory was utilized to assess the associated risks quantitatively.

The findings revealed that the primary types of errors in both loading and unloading operations were action errors and checking errors. Communication errors were also identified in the loading operation. To address these errors, recommendations such as employee training, checklist preparation, installation of audio and visual alarms, and attention to work-rest periods were suggested.

The integration of the SHERPA technique as a qualitative method and the fuzzy inference system as a quantitative method proved to be effective in detecting and quantifying human errors. This approach can assist organizations in allocating resources more efficiently to manage significant risks, thereby enhancing safety and efficiency in petroleum product terminals.

However, it is important to note that this study did not categorize the identified error risks into acceptable and unacceptable levels, which can be a direction for future research. Additionally, incorporating specific criteria for risk categorization and addressing other aspects of human error management would be beneficial for further studies.

Overall, this research contributes to the understanding of human factors in petroleum product terminals and provides insights for improving safety performance. By identifying and addressing human errors, organizations can work towards reducing accidents and enhancing operational effectiveness in the petrochemical industry.

## Informed consent statement

The experts present in the study were fully aware of the work process and expressed their consent to participate in the study in writing.

## Data availability statement

Data, method and references are included in the article.

## CRediT authorship contribution statement

**Mostafa Mirzaei Aliabadi:** Methodology, Investigation. **Iraj Mohammadfam:** Software, Formal analysis. **Samane Khorshidikia:** Writing – review & editing, Writing – original draft, Data curation.

## Declaration of competing interest

The authors declare that they have no known competing financial interests or personal relationships that could have appeared to influence the work reported in this paper.

## References

[1] Shahriari M., Starhe J., Hektor E.J.I.P.V. (2007). Evaluation and IMPROVEMENT of man-machine interaction in tank lorry loading. A CASE STUDY.

[2] Aliabadi M.M., Mohammadfam I., SjjjoHS Khorshidikia (2021). Risk assessment of petroleum products loading arm by BTA technique.

[3] Jemeljanovs V., Sulojeva J., Bartusauskis J., IjsoTE Ingelande (2013).

[4] Furniss D., Sujan M., Henderson J., Embrey D. (2019). 29th European Safety and Reliability Conference ESREL.

[5] Aliabadi M.M., Pourhasan A., Mohammadfam I. (2020). Risk modelling of a hydrogen gasholder using Fuzzy Bayesian Network (FBN). Int. J. Hydrogen Energy.

[6] Rees WJJoE. (1981). Static hazards during the top loading of road tankers with highly insulating liquids: flow rate limitation proposals to minimize risk.

[7] Handayani H.J.J.M. (2018). Implementation of petroleum products tank truck transportation safety management system of company X.

[8] Triana Cedeno G.A. (2000).

[9] Anderson A.R.J.M., Report M.W. (2015). Health effects of cut gas lines and other petroleum product release incidents—seven States, 2010–2012.

[10] Andreyeva E.N., Kryukov V.A. (2008). The Russian model: merging profit and sustainability. Arctic Oil and Gas: Routledge.

[11] Monitoring A. (2007).

[12] Patin S. (2008). Proceedings of International Conference Workshop.

[13] Azadeh A., Mohammad F.I., Garakani M. (2007).

[14] Stirling A.G. (1969). Prevention of Pollution by Oil and Hazardous Materials in Marine Operations. International Oil Spill Conference.

[15] Ghasemi F., Ghasemi A., Kalatpour O. (2022). Prediction of human error probability during the hydrocarbon road tanker loading operation using a hybrid technique of fuzzy sets, Bayesian network and CREAM. Int. J. Occup. Saf. Ergon..

[16] Safaei Y., Dehghan H., Khorshidikia S., Habibi E. (2023). Modeling factors affecting the susceptibility of construction workers to accidents using structural equation model. Int. J. Environ. Health Eng..

[17] Aliabadi M.M., Khorshidikia S. (2020). Evaluation of the possibility of human error in the operation of tower cranes using success likelihood index. Journal of Occupational Hygiene Engineering.

[18] Ghasemi F., Kalatpour O., Moghimbeigi A., Mohammadfam I. (2017). Selecting strategies to reduce high-risk unsafe work behaviors using the safety behavior sampling technique and Bayesian network analysis. J. Res. Health Sci..

[19] Ahmed S., Demirel H.O., Tumer I.Y., Stone R.B.J.T. (2018).

[20] Tsuchiya M., Ikeda H.J.I.P.V. (1991). Human reliability analysis of LPG truck loading operation.

[21] Embrey D. (1986). Proceedings of the International Topical Meeting on Advances in Human Factors in Nuclear Power Systems.

[22] Bjae Kirwan (1992). Human error identification in human reliability assessment. Part 2: detailed comparison of techniques.

[23] Aliabadi M.M., Darvishi E., Shahidi R., Ghasemi F., Mahdinia M. (2020). Explanation and prediction of accidents using the path analysis approach in industrial units: the effect of safety performance and climate. Work.

[24] Rajak A.K., Niraj M., Kumar S. (2016). Designing of fuzzy expert heuristic models with cost management toward coordinating AHP, fuzzy TOPSIS and FIS approaches. Sādhanā.

[25] Li Y., Zhu L. (2020). Risk analysis of human error in interaction design by using a hybrid approach based on FMEA, SHERPA, and fuzzy TOPSIS. Qual. Reliab. Eng. Int..

[26] DjhraL Embrey (2000).

[27] Kirwan B. (2017).

[28] Stanton N.A., Wilson J.A.J.D.C. (2000). Human factors: step change improvements in effectiveness and safety.

[29] Petrillo A., Falcone D., De Felice F., Fjijodrr Zomparelli (2017). Development of a risk analysis model to evaluate human error in industrial plants and in critical infrastructures.

[30] Mirzaei Aliabadi M., Naderi G., Shahtaheri S.J., Forushani A.R., Mohammadfam I., Jahangiri M. (2014). Transport properties of carboxylated nitrile butadiene rubber (XNBR)-nanoclay composites; a promising material for protective gloves in occupational exposures. Journal of Environmental Health Science and Engineering.

[31] Hughes C.M., Baber C., Bienkiewicz M., Worthington A., Hazell A., Hermsdörfer J.J.E. (2015). The application of SHERPA (Systematic Human Error Reduction and Prediction Approach) in the development of compensatory cognitive rehabilitation strategies for stroke patients with left and right brain damage.

[32] Lyons M., Adams S., Woloshynowych M., Vincent C., Si Medicine (2004). Human reliability analysis in healthcare: a review of techniques.

[33] Harris D., Stanton N.A., Marshall A., Young M.S., Demagalski J. (2005). Salmon PJAs, technology. Using SHERPA to predict design-induced error on the flight deck.

[34] Stanton N.A., Salmon P., Harris D., Marshall A., Demagalski J., Young M.S. (2008).

[35] De Felice F., Petrillo A., Zomparelli F.J.I.-P. (2016). A hybrid model for human error probability analysis.

[36] Ung S., Tjoe (2019).

[37] Zimmermann H.-J. (1987).

[38] Gupta P., Kulkarni NJIJoLTiE, Technology (2013). An introduction of soft computing approach over hard computing.

[39] Li Y., Zhu L.J.Q., International R.E. (2020). Risk analysis of human error in interaction design by using a hybrid approach based on FMEA. SHERPA, and fuzzy TOPSIS.

[40] Karimi S., Mirzaei A.M., Mohammad F.I. (2015).

[41] Stanton N., Salmon P., Baber CJe, Alvington (2004).

[42] Lavasani S.M., Zendegani A., Celik M.J.P.S., Protection E. (2015). An extension to Fuzzy Fault Tree Analysis (FFTA) application in petrochemical process industry.

[43] Azadeh A., Rouhollah F., Davoudpour F., Mohammadfam I. (2013). Fuzzy modelling and simulation of an emergency department for improvement of nursing schedules with noisy and uncertain inputs. Int. J. Serv. Oper. Manag..

[44] Ruge B. (2004). Probabilistic Safety Assessment and Management: PSAM 7—ESREL’04 June 14–18, 2004.

[45] Salvi O., Debray B. (2006). A global view on ARAMIS, a risk assessment methodology for industries in the framework of the SEVESO II directive. J. Hazard Mater..

[46] Jamshidi A., Yazdani-Chamzini A., Yakhchali S.H., Sjjolpitpi Khaleghi (2013). Developing a new fuzzy inference system for pipeline risk assessment.

[47] Mamdani E.H., Sjijom-ms Assilian (1975). An experiment in linguistic synthesis with a fuzzy logic controller.

[48] Ramezanifar E., Gholamizadeh K., Mohammadfam I., Aliabadi M., Sa Work (2023). Reliability assessment of fixed foam systems of storage tank based on fuzzy fault tree analysis.

[49] Daftaribesheli A., Ataei M., Sereshki F.J.A.S.C. (2011). Assessment of rock slope stability using the Fuzzy Slope Mass Rating (FSMR) system.

[50] Soltanzadeh A., Sadeghi Yarandi M., Mirzaei Aliabadi M., MjtiiES Mahdinia (2022). Modeling cause-and-effect relationships among predictive variables of human error based on the fuzzy multi-criteria decision-making method.

[51] Mahdinia M., Mohammadfam I., Soltanzadeh A., Aliabadi M.M. (2023). Aghaei HJIjoos, ergonomics. A fuzzy Bayesian network DEMATEL model for predicting safety behavior.

[52] Azariyan H.S., Nikoomaram H., Mirilavasani S.J.O.M. (2022).

[53] Aliabadi M.M., Naderi G., Shahtaheri S.J., Forushani A.R., Mohammadfam I., Jahangiri M. (2014). Mechanical and barrier properties of XNBR-clay nanocomposite: a promising material for protective gloves. Iran. Polym. J. (Engl. Ed.).

[54] Zhou J.-L., Yu Z.-T., Xiao R.-B. (2022). A large-scale group Success Likelihood Index Method to estimate human error probabilities in the railway driving process. Reliab. Eng. Syst. Saf..

